# Supplementary material for: Spin transport in insulators without exchange stiffness
Source: Nat Commun. 2019 Oct 18;10:4740. doi: 10.1038/s41467-019-12749-7 (PMC6800424; doi:10.1038/s41467-019-12749-7)
Supplement: Supplementary file 1 — Supplementary Information [file 41467_2019_12749_MOESM1_ESM.pdf]

Supplementary Information

# Spin transport in insulators without exchange stiffness

Oyanagi et al.

**Table of Contents:**

**Supplementary Note 1. Microscope image of device**

**Supplementary Note 2. Frequency dependence of nonlocal voltage**

**Supplementary Note 3. Theory for spin current at NM/PI interface**

**Supplementary Note 4. Spin current transport in paramagnetic insulator**

**Supplementary Note 5. Spin current transport in the injector and detector electrodes**

**Supplementary Note 6. Nonlocal detector voltage**

**Supplementary Note 7. Magnetic field dependence of the spin diffusion length**

**Supplementary Note 8. Spin current conductance mismatch**

**Supplementary Note 9. Finite element model simulation**

**Supplementary Discussion. Dispersion of dipole spin waves in GGG and YIG**

**Supplementary References**

**Supplementary Note 1.**

**Microscope image of device**

We show a laser microscope image of the nonlocal Pt/GGG/Pt ($d = 2$ μm) device and a schematic illustration of the measurement circuit in Supplementary Fig. 1. The white scale bar corresponds to 50 μm. At the center of the image, two Pt wires (200 μm in length and 100 nm in width) are put on GGG, and they are connected with Pt electrodes.

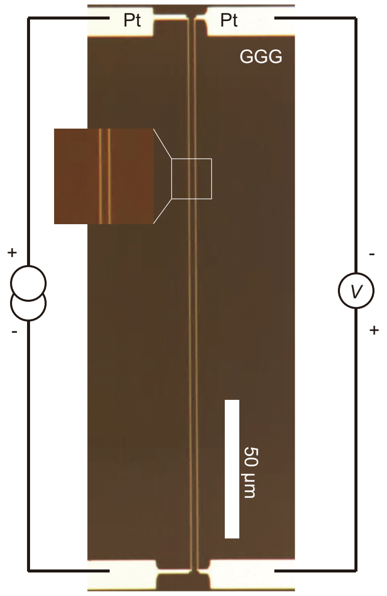


Supplementary Figure 1: Microscope image of nonlocal device. The scale bar shows 50 μm. Two parallel Pt wires are patterned on the top of a GGG slab and connected to a current source and a lock-in amplifier, respectively.

**Supplementary Note 2.**

**Frequency dependence of nonlocal voltage**

We investigated the current frequency $f$ dependence of the nonlocal voltage. Supplementary Figure 2a shows raw data of the magnetic-field angle $\theta$ dependence of the nonlocal voltage $V_{\mathrm{nl}}$ for Pt/GGG/Pt at $B = 3.5$ T and 5 K. A charge current $J_{\mathrm{c}} = 100$ μA at $f = 3.423$ Hz is applied to the Pt injector, and $V_{\mathrm{nl}}$ is measured between the ends of the Pt detector. We can see the modulation of $V_{\mathrm{nl}}$ induced by the variation of $\theta$, following the $\cos^2\theta$ dependence, as discussed in the main text, together with the offset voltage $V_{\mathrm{offset}}$ at $\theta = \pm 90°$. To clarify the origin of the offset voltage, we

measured $V_{nl}$ with applying charge current with various $f$. Supplementary Figure 2b shows the $f$ dependence of $V_{max}$ and $V_{offset}$. We estimated $V_{max}$ and $V_{offset}$ by the fitting $V_{nl} = V_{offset} + V_{max}\cos^2\theta$ to the $\theta$ dependence of $V_{nl}$. The $V_{max}$ signal takes almost a constant value for any $f$ (see also the inset to Supplementary Fig. 2b), being consistent with the expected $f$ dependence of the spin Hall effect (SHE). On the other hand, $V_{offset}$ linearly increases with $f$. The $f$ dependence of $V_{offset}$ indicates a capacitive coupling between the two Pt wires. From the $f$ dependence of $V_{nl}$, we subtracted $V_{offset}$ from $V_{nl}$ and only $V = V_{max}\cos^2\theta$ is shown in the main text to highlight the signal related to the SHE.

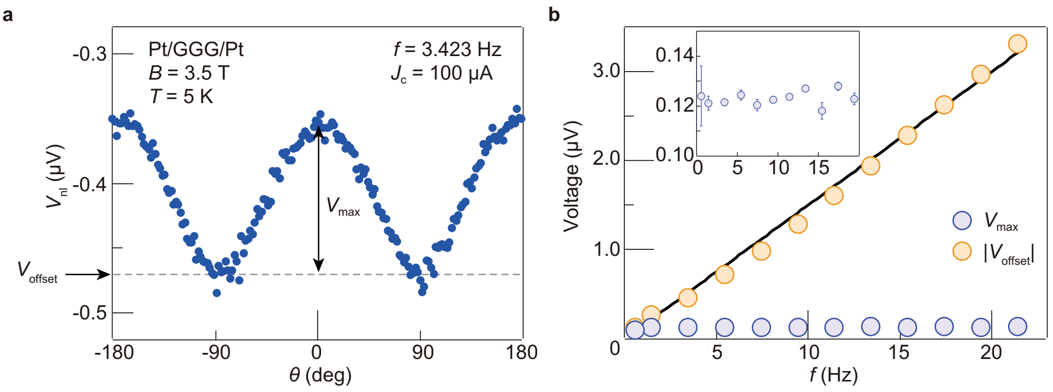


Supplementary Figure 2: Frequency $f$ dependence of nonlocal voltage. **a,** Raw data of the magnetic-field angle $\theta$ dependence of the nonlocal voltage $V_{nl}$. **b,** The $f$ dependence of $V_{max}$ and $V_{offset}$. Blue and yellow plots correspond to $V_{max}$ and $V_{offset}$, respectively. The amplitude of $V_{max}$ and $V_{offset}$ is estimated from the fitting $V_{nl} = V_{offset} + V_{max}\cos^2\theta$ to the raw data. Solid line is for an eye-guide. The inset shows the $f$ dependence of $V_{max}$. The error bars represent the 68% confidence level ($\pm$ s.d.).

**Supplementary Note 3.**

**Theory for spin current at NM/PI interface**

We explain the exchange of spin angular momentum at the interface of normal (paramagnetic) metal (NM)/paramagnetic insulator (PI) by the conventional model[1,2] for the exchange of spin angular momentum at the interface of NM/ferromagnetic insulator (FM). The conduction electrons in Pt and the local paramagnetic moments in GGG are coupled by the interface exchange

interaction[3]. Because the *f*-orbital electrons of $Gd^{3+}$ ion are responsible for the magnetization, this is strictly speaking an *s*-*f* exchange interaction.

Interfacial spin-flip scattering creates and absorbs a non-equilibrium state of localized spin. For example, at the NM/FM interface, spin-flip scattering of a conduction electron in NM creates or absorbs a magnon in FM. In an applied magnetic field, the spin degeneracy of a paramagnetic moment is lifted to split into different energy levels (Supplementary Fig. 3a). Spin-flip scattering causes the transfer of spin angular momentum and energy from a conduction electron to the local moment, of $\pm\hbar$ and the energy by $\pm g\mu_B B$ (Supplementary Fig. 3b), where $\hbar$ is the Dirac's constant, $g$ is the $g$-factor, $\mu_B$ is the Bohr magneton, and $B$ is the applied magnetic field. The spin-flip scattering with an up (down)-spin electron raises (lowers) the magnetic moment at the interface, generating a non-equilibrium state. Assuming weak spin-flip scattering but efficient thermalization, the spin state can be parameterized in terms of a spin chemical potential $\mu_{PI}$ at the lattice temperature. A gradient in the spin chemical potential $\mu_{PI}$ is the driving force of a diffuse

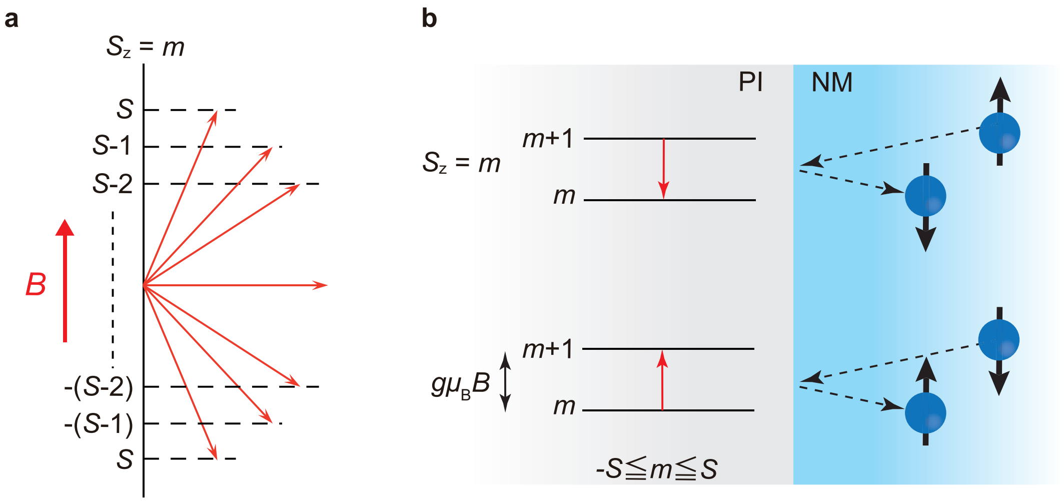


Supplementary Figure 3: Spin-flip scattering at PI/NM interface. **a,** A schematic illustration of Zeeman splitting of a spin-*S* system. Degeneracy is lifted and creates $2S + 1$ energy states by the application of a magnetic field, where the energy splitting is $\Delta E = g\mu_B B$. **b,** A schematic illustration of the spin-flip scattering at an PI/NM interface.

spin current in PI.

The *s*-*f* exchange $\mathcal{J}_{\mathrm{sf}}$ governs the interaction between magnetic moments of PI and conduction electrons at the interface

$$\widehat{\mathcal{H}}_{\mathrm{sf}} = -\, v_{\mathrm{N}} \mathcal{J}_{\mathrm{sf}} \sum_{n=1}^{N_{\mathrm{PI}}} \widehat{\boldsymbol{S}}_n(t) \cdot \widehat{\boldsymbol{\sigma}}(\boldsymbol{r}_n), \tag{1}$$

where $\widehat{\boldsymbol{S}}_n$ is the local spin at the position $r_n$, $\widehat{\boldsymbol{\sigma}}(\boldsymbol{r}_n)$ is the conduction electron spin density at $r_n$, $v_{\mathrm{N}}$ is the unit cell volume of NM, and $N_{\mathrm{PI}}$ is the number of local spins at the interface.

The spin-current density operator at the interface $\widehat{j}_{\mathrm{s}}(t) = (\hbar/2A)\, \mathrm{d}(\widehat{N}_e^{\uparrow} - \widehat{N}_e^{\downarrow})/\mathrm{d}t$ ($\widehat{N}_e^{\sigma}$ is the number operator of conduction electrons with spin $\sigma$ and $A$ is the area size of the interface) is calculated by the Heisenberg equation of motion as

$$\widehat{j}_{\mathrm{s}}^{z} = -\frac{v_{\mathrm{N}} \mathcal{J}_{\mathrm{sf}}}{A} \sum_{n=1}^{N_{\mathrm{PI}}} \left[\widehat{\boldsymbol{S}}_n(t) \times \widehat{\boldsymbol{\sigma}}(\boldsymbol{r}_n)\right]_z . \tag{2}$$

The second-order perturbation of the NM by $\mathcal{J}_{\mathrm{sf}}$ allows us to obtain the spin current density $j_{\mathrm{s}}^{\mathrm{N/PI}}$ through the interface as

$$j_{\mathrm{s}}^{\mathrm{N/PI}} = -\frac{i}{\hbar} \int_{-\infty}^{t} \mathrm{d}t' \langle [\widehat{J}_{\mathrm{s}}(t'), \widehat{\mathcal{H}}_{\mathrm{sf}}(t')] \rangle$$

$$= \frac{n_{\mathrm{PI}}}{\hbar} (\mathcal{J}_{\mathrm{sf}} v_{\mathrm{N}})^2 \int_{-\infty}^{\infty} \mathrm{d}t \sum_p \left[ \langle \widehat{S}_+(t) \widehat{S}_-(0) \rangle \langle \widehat{\sigma}_{-p}^{-}(t) \widehat{\sigma}_p^{+}(0) \rangle - \langle \widehat{S}_-(t) \widehat{S}_+(0) \rangle \langle \widehat{\sigma}_p^{+}(t) \widehat{\sigma}_{-p}^{-}(0) \rangle \right], \tag{3}$$

where $n_{\mathrm{PI}} = N_{\mathrm{PI}}/A$ is the areal density of the number of local spins at the interface, $\widehat{S}_{\pm} = \widehat{S}_x \pm i\widehat{S}_y$, and $\widehat{\sigma}^{\pm} = \widehat{\sigma}_x \pm i\widehat{\sigma}_y$.

Using the fluctuation-dissipation theorem for the correlation functions in Supplementary Eq. (3),

$$j_{\mathrm{s}}^{\mathrm{N/PI}} = 2 j_{\mathrm{s}}^{0} \frac{g\mu_{\mathrm{B}} S B - \mu_{\mathrm{N}}}{k_{\mathrm{B}} T_{\mathrm{N}}} B_s \left( \frac{g\mu_{\mathrm{B}} S B_{\mathrm{eff}}}{k_{\mathrm{B}} T_{\mathrm{PI}}} \right) \times$$

$$\left[n_{\rm B}\left(g\mu_{\rm B}B_{\rm eff}-\mu_{\rm PI},T_{\rm PI}\right)-n_{\rm B}\left(g\mu_{\rm B}B-\mu_{\rm N},T_{\rm N}\right)\right], \quad (4)$$

where $j_{\rm s}^{0}=4\pi n_{\rm PI}S[\mathcal{J}_{\rm sf}v_{\rm N}N(0)]^{2}(k_{\rm B}T_{\rm N})$, $n_{\rm B}$ is the Bose distribution function, $N(0)$ is the density of states at the Fermi level, $T_{\rm N}$ is the temperature of NM, $T_{\rm PI}$ is the temperature of PI, $B_s(x)$ is the Brillouin function as a function of $x$ for spin-$S$, $B_{\rm eff}=[T_{\rm PI}/(T_{\rm PI}+|\Theta_{\rm CW}|)]B$ is the Curie-Weiss molecular magnetic field[4] in GGG, $\Theta_{\rm CW}$ is the Curie-Weiss temperature of GGG, and $\mu_{\rm N}$ is the spin accumulation in NM at the interface. For $\delta\ll 1$, $n_{\rm B}(x-\delta)\sim(1/2)[(\delta/\sinh^{2}x)-1]$ and Supplementary Eq. (4) simplifies to

$$j_{\rm s}^{\rm N/PI}\approx\frac{\hbar}{2e^{2}}g_{\rm s}(\mu_{\rm PI}-\mu_{\rm N}), \quad (5)$$

where $g_{\rm s}=(4\pi n_{\rm PI}e^{2}/\hbar)S[\mathcal{J}_{\rm sf}v_{\rm N}N(0)]^{2}(\xi/\sinh^{2}\xi)B_{s}(2S\xi)$ is the effective interface spin conductance, $\xi=(g\mu_{\rm B}B_{\rm eff}/2k_{\rm B}T)$, and $e$ is the element charge.

In this formalism, the interface spin current is proportional to the difference in the non-equilibrium parameters of the spins on the NM and PI sides, as for the NM/FM interface for which $\mu_{\rm PI}$ is replaced by the magnon chemical potential. On the other hand, the effective interface spin conductance $g_{\rm s}$ is here quite different from that of the NM/FM interface. The much stronger magnetic field and temperature dependence reflect the need to generate magnetic order by the Zeeman interaction, which exists spontaneously in the FM.

**Supplementary Note 4.**

**Spin current transport in paramagnetic insulator**

In our model, we assume that spin current in a paramagnetic insulator can be described by a diffusion process, which explains transport of conduction-electron spin current in a metal[5] and magnon spin current in a magnetic insulator[6]. Through the interface exchange coupling, spin current is injected into or absorbed by a metal, creating a non-equilibrium spin accumulation $\mu_{\rm PI}$ in the

(para)magnetic insulator. The diffusion equations on both sides with appropriate boundary conditions govern the spatial profiles of $\mu_{\mathrm{PI}}$ and the spin current.

Supplementary Figure 4a shows a schematic side view of the Pt/GGG/Pt. The injector and the detector electrodes are placed at $(x,y) = (0,0)$ and $(d,0)$ on the surface of GGG, respectively. Because the thickness of the GGG of 500 μm is much larger than the distance $d$ (~ μm) between the electrodes, we have to use the two-dimensional diffusion equation:

$$\left(\frac{1}{\lambda_{\mathrm{PI}}^2} - \partial_x^2 - \partial_y^2\right)\mu_{\mathrm{PI}} = 0, \tag{6}$$

where $\lambda_{\mathrm{PI}}$ is the diffusion length (or the relaxation length) of spin current in PI. Injected spin current flows away radially from the injector and is absorbed radially by the detector as shown in Supplementary Fig. 4a. When $d$ is much larger than the contact size $w$, the solution of the two-dimensional diffusion equation, Supplementary Eq. (6), reads

$$\mu_{\mathrm{PI}}(x,y) = aK_0\left(r/\lambda_{\mathrm{PI}}\right) - bK_0\left(|\mathbf{r} - \mathbf{d}|/\lambda_{\mathrm{PI}}\right), \tag{7}$$

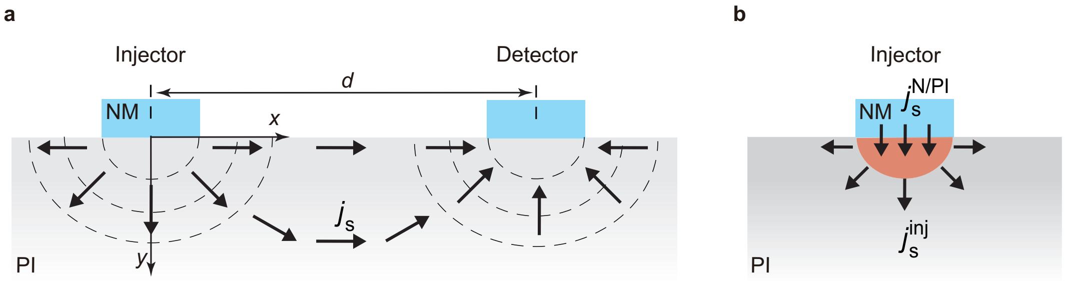


Supplementary Figure 4: Two-dimensional spin-current flow in PI. **a,** A schematic side view of the sample geometry. The left NM contact creates a spin accumulation in the PI. Arrows represent the spin-current flow, driven by the gradient of $\mu_{\mathrm{PI}}$ in PI that flows radially away from the injector and is (partially) collected by the detector. **b,** A magnified view of the injector. The SHE spin current $J_s^{N/PI}$ flows normal to the interface, while $J_s^{inj}$ expands radially in the PI. When the semi-circular region indicated by the red shading is much smaller than the spin-flip length, both spin currents are related by a geometrical factor.

where $K_0\,(r/\lambda_{\mathrm{PI}})$ is the modified Bessel function of the second kind, $r = |\mathbf{r}| = \sqrt{x^2+y^2}$, and $a$ and $b$ are the coefficients that are determined by the boundary conditions that spin currents are continuous at the contacts between PI and NM. In PI, spin current density is given by

$$j_{\mathrm{s}}^{\mathrm{PI}} = -\frac{\hbar}{2e^2}\sigma_{\mathrm{PI}}\nabla\mu_{\mathrm{PI}}, \tag{8}$$

where $\sigma_{\mathrm{PI}} = e^2 n_{\mathrm{s}} D_{\mathrm{PI}}/(k_{\mathrm{B}} T_{\mathrm{PI}} \sinh^2\xi)$ is the spin conductivity of PI and $n_{\mathrm{s}}$ is the density of localized spins. The diffusion constant $D_{\mathrm{PI}}$ is governed by the physics of the diffusion process and depends on $B$ and $T$. The experiments are fitted by $D_{\mathrm{PI}} \sim 10^{-9}$ m$^2$/s which is orders of magnitude larger than the diffusion induced by the incoherent dipole-dipole interaction[7]. The efficient spin transport can be explained in terms of collective magnetostatic spin waves with a group velocity that roughly scales with the field-induced magnetization $v \sim M(B,T)$. The spin conductivity then scales like $\sigma_{\mathrm{PI}} \sim (1 - M(B,T)/M_{\mathrm{s}})[M(B,T)/M_{\mathrm{s}}]^2$, where $M_{\mathrm{s}}$ is the paramagnetic saturation magnetization, but in the absence of a microscopic model we take it as an adjustable parameter.

**Supplementary Note 5.**

**Spin current transport in the injector and detector electrodes**

A charge current density $j_{\mathrm{c}}$ in the injector electrode ($z$ direction) induces a transverse spin current density in the $y$ direction according to

$$j_{\mathrm{s}}^{\mathrm{N}}\,(y) = -\frac{\hbar}{2e^2}\sigma_{\mathrm{N}}\nabla\mu_{\mathrm{N}}\,(y) + \frac{\hbar}{2e}\theta_{\mathrm{SH}} j_{\mathrm{c}}, \tag{9}$$

where $\sigma_{\mathrm{N}}$ is the charge conductivity and $\theta_{\mathrm{SH}}$ is the spin Hall angle. The spin accumulation $\mu_{\mathrm{N}}(y)$ satisfies the diffusion equation

$$\nabla^2\mu_{\mathrm{N}}\,(y) = \frac{\mu_{\mathrm{N}}\,(y)}{\lambda_{\mathrm{N}}^2}, \tag{10}$$

where $\lambda_N$ is the spin diffusion length. Supplementary Equation (10) has a solution of the form

$$\mu_N(y) = \frac{2e^2}{\hbar}\frac{2\lambda_N}{\sigma_N}\frac{\cosh[(y-t_N)/\lambda_N]}{\sinh(t_N/\lambda_N)} j_s^{N/PI} - \frac{2e}{\sigma_N}\theta_{SH}\lambda_N \frac{\sinh[(2y-t_N)/2\lambda_N]}{\cosh(t_N/2\lambda_N)} j_c, \quad (11)$$

where $t_N$ is the thickness of NM.

By combining Supplementary Eq. (5) with Supplementary Eq. (11), the interface spin current density becomes

$$j_s^{N/PI} \approx \frac{\hbar}{2e^2} g_s \mu_{PI} - \frac{\hbar}{2e}\frac{\lambda_N}{\sigma_N}\theta_{SH} g_s \tanh(t_N/2\lambda_N) j_c. \quad (12)$$

The spin accumulation and spin current density at the detector are obtained analogously, where we set $j_c = 0$ in the injector. The electric field induced by the ISHE in the detector depends on the absorbed spin current density $j_s^{PI/N}$ as

$$E_{ISHE} = (\theta_{SH}/\sigma_N)(\lambda_N/t_N)(2e/\hbar) j_s^{PI/N} \tanh(t_N/2\lambda_N). \quad (13)$$

**Supplementary Note 6.**

**Nonlocal detector voltage**

Here we derive the voltage over the detector electrode for spin current-conserving boundary conditions at the contacts, i.e. $j_s^{PI}$ in PI and $j_s^N$ in the injector and detector are related by the interface spin currents density $j_s^{N/PI}$ between PI and NM. As shown in Supplementary Fig. 4b, at the NM/PI interface, the interfacial spin current density $j_s^{N/PI}$ and metallic spin current density $j_s^N$ flow normal to the interface. On the other hand, for sufficiently narrow contacts, the spin current in PI near the injector flows in a semi-circular pattern with amplitude $j_s^{inj}$ as indicated in Supplementary Fig. 4b, while $j_s^{det}$ near the detector is absorbed. We assume that the spin current density flows normal to the interface and a radially symmetric spin current densities in the PI are conserved in the red region

of Supplementary Fig. 4b such that

$$\pi r_0 j_s^{\rm int} = -\, w_{\rm N} j_s^{\rm N/PI},\ \pi r_0 j_s^{\rm det} = -\, w_{\rm N} j_s^{\rm PI/N}, \tag{14}$$

where $\pi r_0$ is the circumference of the semi-circle of radius $r_0$ and $w_{\rm N}$ is the contact width. We adopt $w_{\rm N}/2 = r_0$ as shown in Supplementary Fig. 4b such that

$$j_s^{\rm inj} = -\frac{\hbar}{2e^2}\sigma_{\rm PI}\left[\nabla\mu_{\rm PI}\right]_{r=r_0},\ j_s^{\rm det} = -\frac{\hbar}{2e^2}\sigma_{\rm PI}\left[\nabla\mu_{\rm PI}\right]_{|r-d|=r_0}. \tag{15}$$

With these conditions we arrive at a nonlocal voltage $V$ induced by the ISHE in the detector as

$$V = C_0\frac{\Lambda_1 K_0\,(d/\lambda_{\rm PI})}{[1+\Lambda_2 K_0\,(r_0/\lambda_{\rm PI})]^2 - [\Lambda_2 K_0\,(d/\lambda_{\rm PI})]^2}, \tag{16}$$

where

$C_0 = (\theta_{\rm SH}^2 L_{\rm N} J_{\rm c} \lambda_{\rm N}/\sigma_{\rm N} t_{\rm N})\tanh^2(t_{\rm N}/2\lambda_{\rm N})$,

$\Lambda_1 = (\sigma_{\rm N} w_{\rm N}/2\pi\lambda_{\rm N}\sigma_{\rm PI})\left[2\rho_{\rm N}\lambda_{\rm N} g_{\rm s}/(1+2\rho_{\rm N}\lambda_{\rm N} g_{\rm s}\coth(t_{\rm N}/\lambda_{\rm N}))\right]^2 \approx (2w_{\rm N}\lambda_{\rm N}/\pi\sigma_{\rm N})(g_{\rm s}^2/\sigma_{\rm PI})$, and

$\Lambda_2 = (\sigma_{\rm N} w_{\rm N}/2\pi\lambda_{\rm N}\sigma_{\rm PI})\left[2\rho_{\rm N}\lambda_{\rm N} g_{\rm s}/(1+2\rho_{\rm N}\lambda_{\rm N} g_{\rm s}\coth(t_{\rm N}/\lambda_{\rm N}))\right] \approx (w_{\rm N}/\pi)(g_{\rm s}/\sigma_{\rm PI})$

for small spin conductance ($\rho_{\rm N}\lambda_{\rm N} g_{\rm s} \ll 1$), which is always the case at sufficiently low temperatures.

When $d$ is larger than $\lambda_{\rm PI}$, $[\Lambda_2 K_0(d/\lambda_{\rm PI})]^2$ in Supplementary Eq. (16) can be disregarded, so that Supplementary Eq. (16) reduces to equation (1) in the main text,

$$V_{\rm max}(d) \approx C K_0(d/\lambda_{\rm PI}), \tag{17}$$

where $C = C_0\Lambda_1/[1+\Lambda_1 K_0(r_0/\lambda_{\rm PI})]^2$.

The $d$ dependence of $V_{\rm max}$ for $\lambda_{\rm PI=GGG}$ in the range from 0.5 μm to 10 μm is plotted in

Supplementary Fig. 5a. The red line shows the best fit with $\lambda_{GGG} = (1.82 \pm 0.19)$ μm and $C = (76.45 \pm 5.89)$ nV.

A one-dimensional spin diffusion model predicts simple exponential decay of $V_{max}$. In Supplementary Fig. 5b, we fit $V_{max} = C\exp(-d/\lambda_{GGG})$ to the data with very different $\lambda_{GGG}$ (1D) = (0.98 ± 0.07) μm, which indicates a significant diffusion via paths deep in the sample.

Next, we address equation (2) in the main text for $\xi' = \xi(B = 3.5\ \mathrm{T}, T = 5\ \mathrm{K})$, where $\xi = (g\mu_B B_{eff}/2k_B T)$ and $B_{eff} = [T_{PI}/(T_{PI} + |\Theta_{CW}|)]B$. The spin conductivity $\sigma_{PI}$ and the interface spin conductance $g_s$ can be written as $\sigma_{PI} = e^2 n_s D_{PI}/k_B T \sinh^2\xi = \sigma_{PI}(\xi')\sinh^2\xi'/\sinh^2\xi$ and $g_s = 4\pi n_{PI} e^2 S[J_{sf}\nu_N N(0)]^2 \xi B_s(2S\xi)/(\hbar \sinh^2\xi) = g_s(\xi')(\xi/\xi')(\sinh^2\xi'/\sinh^2\xi)(B_s(2S\xi)/B_s(2S\xi'))$ , respectively. Supplementary Equation (16) can be rewritten in terms $\sigma_{PI}(\xi')$ and $g_s(\xi')$ and equation (2) in the main text is obtained,

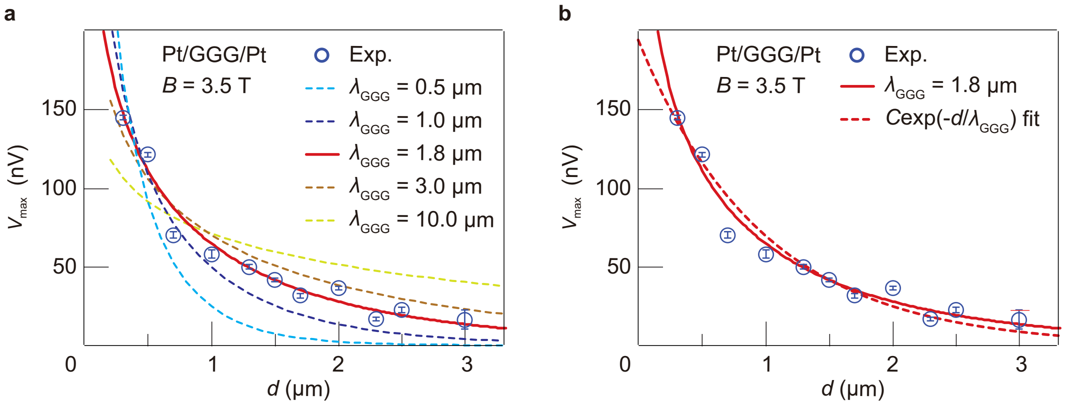


Supplementary Figure 5: $d$ dependences of $V$ with various $\lambda_{GGG}$. Experimental (blue plots) and calculated (lines) $V_{max}$ as a function of $d$. Experiments are carried out at 5 K and 3.5 T. **a.** The red curve is the best fit by Supplementary Eq. (17), $V_{max}(d) = CK_0(d/\lambda_{GGG})$ with $\lambda_{GGG} = 1.8$ μm. The dashed curves are fits for fixed $\lambda_{GGG}$, varied from 0.5 μm to 10 μm. **b.** The red and dashed curves are the best fit by Supplementary Eq. (17), and the exponential fit with $\lambda_{GGG} = 0.98$ μm, respectively.

$$V(B,T) = C_1 \frac{g_s^2(\xi')}{\sigma_{PI}(\xi')} \frac{[(\xi)B_s(2S\xi)/\sinh(\xi)]^2}{[1 + C_2 \frac{g_s(\xi')}{\sigma_{PI}(\xi')} \xi B_s(2S\xi)]^2}, \quad (18)$$

where

$C_1 = C_0(2w_N\lambda_N/\pi\sigma_N)(\sinh^2\xi'/\xi' B_s(2S\xi'))^2 K_0(d/\lambda_{PI})$ and

$C_2 = (2w_N\lambda_N/\pi\sigma_N)(K_0(r_0/\lambda_{PI})/\xi' B_s(2S\xi'))$.

In our samples NM = Pt and PI = GGG with the contact distance $d = 0.5$ μm, contact width $w_{Pt} = 100$ nm, $r_0 = w_{Pt}/2 = 50$ nm, $t_{Pt} = 10$ nm, $L_{Pt} = 200$ μm, $J_c = 100$ $\mu A_{rms}$, and $T = 5$ K. We use the material parameters[6]: $\lambda_{GGG} = 1.82$ μm, $\lambda_{Pt} = 1.5$ nm, $\theta_{SH} = 0.11$, $\sigma_{Pt} = 3.4 \times 10^4$ $Sm^{-1}$, $\Theta_{CW} = -2$ K, $g = 2$, $S = 7/2$ for the fit of Supplementary Eq. (18) to the measured $B$ dependence of $V$. As discussed in the main text, we estimated $\sigma_{GGG}(B = 3.5\text{ T}) = (7.25 \pm 0.26) \times 10^4$ $Sm^{-1}$ and $g_s(B = 3.5\text{ T}) = (1.82 \pm 0.05) \times 10^{11}$ $Sm^{-2}$.

We show the magnetic field dependence of $\sigma_{GGG} = e^2 n_s D_{GGG}/k_B T \sinh^2\xi = \sigma_{GGG}(\xi')\sinh^2\xi'/\sinh^2\xi$ and

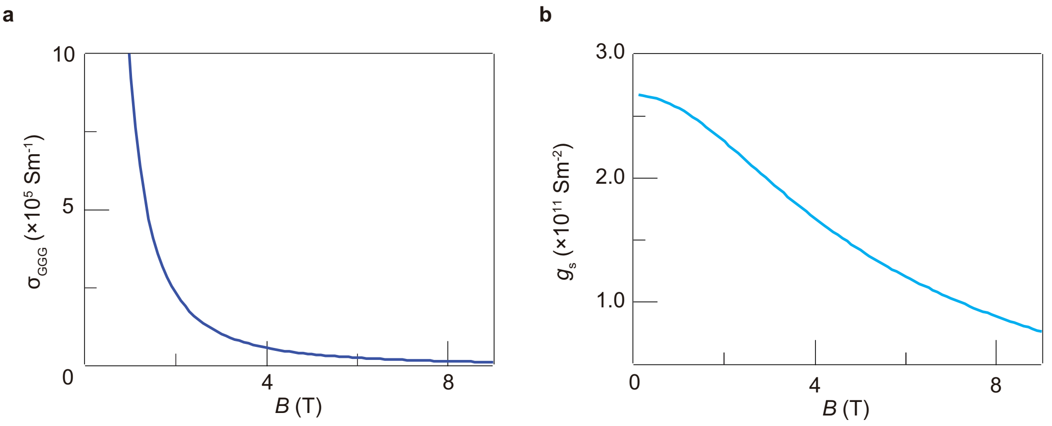


Supplementary Figure 6: $B$ dependences of $\sigma_{GGG}$ and $g_s$. **a,** The magnetic field $B$ dependence of the spin conductivity $\sigma_{GGG} = \sigma_{GGG}(\xi')\sinh^2\xi'/\sinh^2\xi$, where $\xi' = \xi(B = 3.5\text{ T}, T = 5\text{ K})$ and $\sigma_{GGG}(\xi') = 7.2 \times 10^4$ $Sm^{-1}$. **b,** $B$ dependence of the effective spin conductance $g_s = g_s(\xi')(\xi/\xi')(\sinh^2\xi'/\sinh^2\xi)(B_s(2S\xi)/B_s(2S\xi'))$, where $g_s(\xi') = 1.82 \times 10^{11}$ $Sm^{-2}$. $\sigma_{GGG}$ and $g_s$ are calculated in the magnetic-field range from 0.2 T to 9 T.

$g_s = 4\pi n_{PI} e^2 S[\mathcal{J}_{sf} v_N N(0)]^2 \xi B_s(2S\xi)/(\hbar \sinh^2\xi) = g_s(\xi')(\xi/\xi')(\sinh^2\xi'/\sinh^2\xi)(B_s(2S\xi)/B_s(2S\xi'))$ , respectively, in Supplementary Fig. 6. Here, $\sigma_{GGG}(\xi') = 7.25 \times 10^4$ Sm$^{-1}$ and $g_s(\xi') = 1.82 \times 10^{11}$ Sm$^{-2}$.

Supplementary Figure 6a shows the $B$ dependence of $\sigma_{GGG}$ that diverges at zero field since the diffusion constant $D_{GGG}$ does not vanish in this limit (here we take the latter independent of $B$). On the other hand, $g_s$ in Supplementary Fig. 6b depends only weakly on the magnetic field.

Supplementary Figure 7 shows the best fit of Supplementary Eq. (18) to the $B$ dependence of $V$ with $d$ = 0.5 μm, 1.0 μm, and 3.0 μm. As discussed in the main text, Supplementary Eq. (18) reproduces the observations. The best fits for $d$ = 1.0 μm and $d$ = 3 μm are obtained slightly different parameters, i.e. $\sigma_{GGG}(\xi') = (4.35 \pm 0.36) \times 10^4$ Sm$^{-1}$ and $g_s(\xi') = (1.33 \pm 0.09) \times 10^{11}$ Sm$^{-2}$, and $\sigma_{GGG}(\xi') = (4.20 \pm 0.72) \times 10^4$ Sm$^{-1}$ and $g_s(\xi') = (1.77 \pm 0.24) \times 10^{11}$ Sm$^{-2}$, respectively. The parameters for $d$ = 1.0 μm and 3.0 μm do not much differ from those for $d$ = 0.5 μm ($\sigma_{GGG}(B = 3.5\text{ T}) = (7.25 \pm 0.26) \times 10^4$ Sm$^{-1}$ and $g_s(B = 3.5\text{ T}) = (1.82 \pm 0.05) \times 10^{11}$ Sm$^{-2}$). We conclude that our theoretical model can explain the $B$ dependence of $V$ for all $d$ for very similar

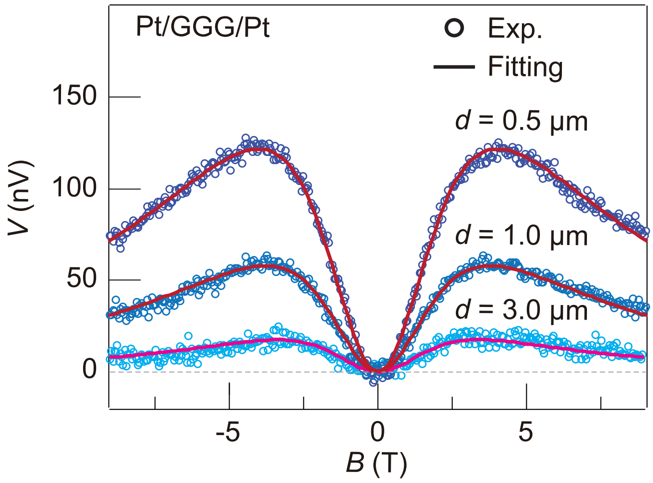


Supplementary Figure 7: $B$ dependences of $V$ with different $d$. The experimental and fitting results of $V$ as a function of $B$ with different values of $d$. The plots represent the experimental results of $d$ = 0.5, 1.0, and 3.0 μm. We fit Supplementary Eq. (18) to each experimental results, shown as solid curves.

$\sigma_{GGG}$ and $g_s$.

**Supplementary Note 7.**

**Magnetic field dependence of the spin diffusion length**

We extract the $B$ dependence of $\lambda_{GGG}$ at 5 K by fitting Supplementary Eq. (17) to the data in Supplementary Fig. 7. We observe in Supplementary Fig. 8 that by increasing $B$ from 0, $\lambda_{GGG}$ increases with a broad maximum at around 2 - 4 T. A finite magnetization is beneficial for the spin diffusion in GGG, but only up to a degree. By further increasing $B$, $\lambda_{GGG}$ gradually decreases, consistent with a previous report on YIG[8] and presumably related to magnon freeze out.

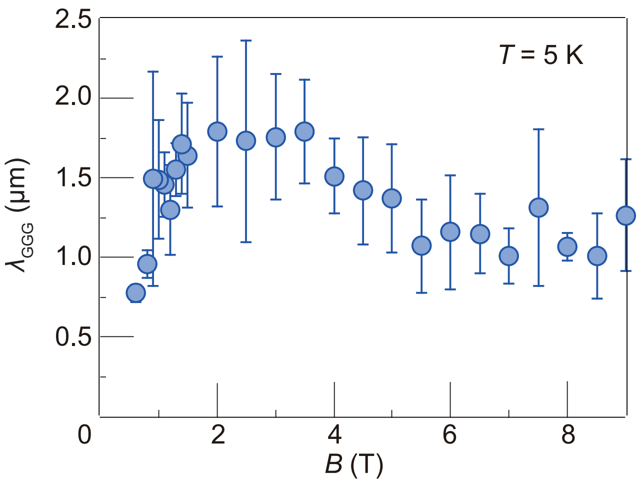


Supplementary Figure 8: $B$ dependence of $\lambda_{GGG}$. $\lambda_{GGG}$ is estimated by a fit of $V_{nl}(d) = CK_0(d/\lambda_{GGG})$ to the $d$ dependence of $V_{nl}$. The error bars represent the 68 % confidence level (± s.d.).

**Supplementary Note 8.**

**Spin current conductance mismatch**

Here we briefly discuss the physical origin of the vanishing voltage signal $V$ at zero magnetic field at the hand of the one-dimensional model for spin transport in a NM/PI/NM trilayer structure in Supplementary Fig. 9. We calculate spin currents at the spin injector and detector interfaces at $x = 0$ and $L$, respectively as explained in Supplementary Note 3-6 to obtain the spin current density at the injector NM/PI and detector PI/NM interfaces

$$j_s^{N/PI}(0) = -\frac{\hbar}{2e}\theta_{SH}j_c \frac{g_N}{1+g_{PI}} \frac{\tanh(t_N/2\lambda_N)}{1-[(1-g_{PI})/(1+g_{PI})]^2\exp(-2L/\lambda_{PI})}\left[1 - \frac{1-g_{PI}}{1+g_{PI}}\exp(-2L/\lambda_{PI})\right], \quad (19)$$

$$j_s^{PI/N}(L) = -\frac{\hbar}{2e}\theta_{SH}j_c \frac{g_N}{1+g_{PI}} \frac{g_{PI}}{1+g_{PI}} \frac{2\tanh(t_N/2\lambda_N)\exp(-L/\lambda_{PI})}{1-[(1-g_{PI})/(1+g_{PI})]^2\exp(-2L/\lambda_{PI})}, \quad (20)$$

where $g_N = 2g_s\lambda_N/\sigma_N$ and $g_{PI} = 2g_s\lambda_{PI}/\sigma_{PI}$, respectively. The dependence of the interface spin currents $j_s^{N/PI}(0) \propto \frac{g_N}{1+g_{PI}}$ and $j_s^{PI/N}(L) \propto \frac{g_{PI}}{1+g_{PI}}$ agrees with results from Supplementary Note 3-6 except for numerical constants.

For PI = GGG, $g_{PI} \sim 1/\sigma_{PI}$ vanishes with the applied field, see Supplementary Fig. 6a, while $j_s^{N/PI}(0)$ is maximal. The spin angular momentum induced via the SHE in NM is efficiently injected into the PI because in this limit the Zeeman gap (see Supplementary Fig. 3b) and backflow spin current vanish.

On the other hand, at the detector $j_s^{PI/N}(L)$ vanishes with $g_{PI}$. The spin current cannot enter the NM from the PI with a large spin conductivity because the dissipation of the spin accumulation in the PI

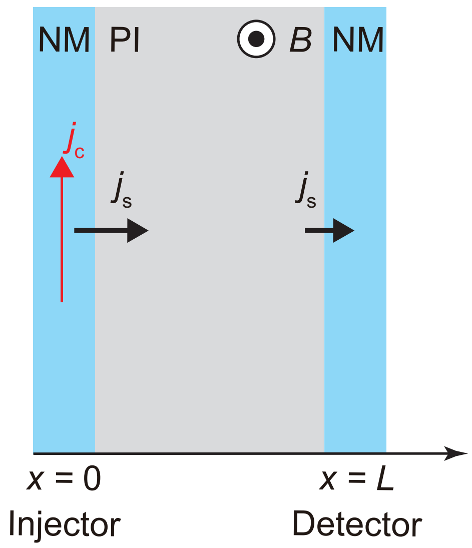


Supplementary Figure 9: One-dimensional model. Schematic NM/PI/NM trilayer structure to illustrate the spin current conductance mismatch. The black and red arrows indicate the direction of spin and charge currents, respectively.

is strongly enhanced when it cannot escape easily into the contact. The difficulty of spin injection from a metal with a high to one with a low conductivity is known as the impedance-mismatch problem[9].

**Supplementary Note 9.**

**Finite element model simulation**

We also calculate spin transport in GGG numerically by a finite element model (FEM) to check the consistency with our analytical model and validate the approximations of the geometry. This method as used to describe magnon spin transport in YIG (in Supplementary Refs 6, 10) can be applied as well to the present problem since the new physics is reflected in the phenomenological parameters. A two-dimensional diffusion equation is FEM-implemented with COMSOL multi-physics (v4.4), yielding the chemical potential profile $\mu_{GGG}$ and the nonlocal voltage $V$ at the detector. The sample geometry is close to the experiments (i.e., two Pt wires with thickness of 10

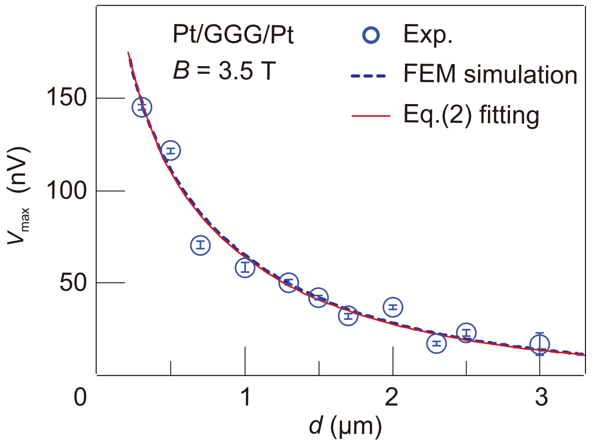


Supplementary Figure 10: FEM result for $d$ dependence of $V_{max}$. The comparison between the results of the FEM, our analytical model, and the experiment for the nonlocal voltage as a function of $d$. The blue plots show the experimental results of the $d$ dependence of $V_{max}$ at 5 K with applying magnetic field of 3.5 T. The blue dotted curve shows the result of the FEM with the input of $\sigma_{GGG} = 7.25 \times 10^4$ Sm$^{-1}$ and $g_s = 1.82 \times 10^{11}$ Sm$^{-2}$. The red curve shows the best fitting of equation (2) = Supplementary Eq. (17). The error bars represent the 68% confidence level (± s.d.).

nm and width of 100 nm on top of a GGG slab with the thickness of 500 μm and width of 10 mm) with material parameters listed in the previous section. The distance of the Pt wires are varied from $d = 0.3$ μm to 3.0 μm. The nonlocal voltage depends on two free parameters, $g_s$ of the Pt/GGG interface and $\sigma_{GGG}$ of GGG.

We calculated the $d$ dependence of $V_{max}$ with the parameter values obtained from our analytical model. Supplementary Figure 10 shows the FEM simulation result with $\sigma_{GGG} = 7.25 \times 10^4\ \mathrm{Sm^{-1}}$ and $g_s = 1.82 \times 10^{11}\ \mathrm{Sm^{-2}}$ as the blue dotted curve. In Supplementary Fig. 10, we also show the result of our analytical model with the same parameter values, the best fit of Supplementary Eq. (17), as a red solid curve. The FEM simulations are consistent with our analytical model for the entire distance range.

We can also try to fit the data in FEM. We fixed $g_s$ as the value estimated using our analytical model ($g_s = 1.82 \times 10^{11}\ \mathrm{Sm^{-2}}$) and introduced one free parameter $\sigma_{GGG}$ with an initial guess of $7.25 \times 10^4\ \mathrm{Sm^{-1}}$. We found a best fit of the FEM model for $\sigma_{GGG} = (6.98 \pm 0.8) \times 10^4\ \mathrm{Sm^{-1}}$. This is consistent with that estimated using our analytical model ($\sigma_{GGG} = (7.25 \pm 0.26) \times 10^4\ \mathrm{Sm^{-1}}$). The agreements between the two models indicate that Supplementary Eq. (17) is consistent with the numerical calculation, i.e. the simplifications of the geometry that allow the analytic treatment, are justified for the present sample dimensions.

**Supplementary Discussion.**

**Dispersion on dipole spin wave in GGG and YIG**

We compute spin wave dispersion of GGG and YIG following Supplementary Ref. 11. In both YIG and GGG the long-range dipole interaction dominates the spin wave dispersion. Their frequencies are governed by the dipolar-gap plus the Zeeman energy[11] $hf = g\mu_B\sqrt{B(B + 4\pi M_0(1 - \exp(-kt))/kt)}$, where $M_0$ is the magnetization, $t$ is the thickness, and $k$

is the wave number, as discussed below. Supplementary Figures 11a and 11b respectively show the spin-wave dispersions at $T = 5$ K at several magnetic fields and the spin-wave gap (at $k = 0$) as a function of $B$ (dashed lines indicate $hf = k_B T$); below (above) these lines, thermal occupation is large (small). In the present nonlocal configuration the wave vector **k** is parallel to the magnetic field **B** and transport is mainly carried by backward volume magnetostatic waves (BVMSW).

In GGG the frequency $f$ decreases with increasing wave number $k$ and saturates at a constant value for $k > 10^3$ rad/cm. While spin waves with frequencies up to the spin accumulation induced by the spin Hall effect (SHE) in the injector Pt wire are excited, only the thermally occupied ones contribute to long-range transport. The spin current in GGG is therefore conveyed only by the spin waves with significant group velocities close to $k = 0$ ($k < 10^3$ rad/cm). Their frequencies are comparable to the frequency gap determined by the dipole and Zeeman interactions: For $T = 5$ K, the relevant spin-wave frequencies are from ~ 28 to 37 GHz at $B = 1$ T, from ~ 97 to 108 GHz at $B = 3.5$ T, and from ~ 224 to 234 GHz at $B = 8$ T.

The exchange stiffness in YIG (roughly) adds a parabolic contribution to the dipolar dispersion. Supplementary Figure 11c compares the spin-wave band structures for YIG (red solid lines) and

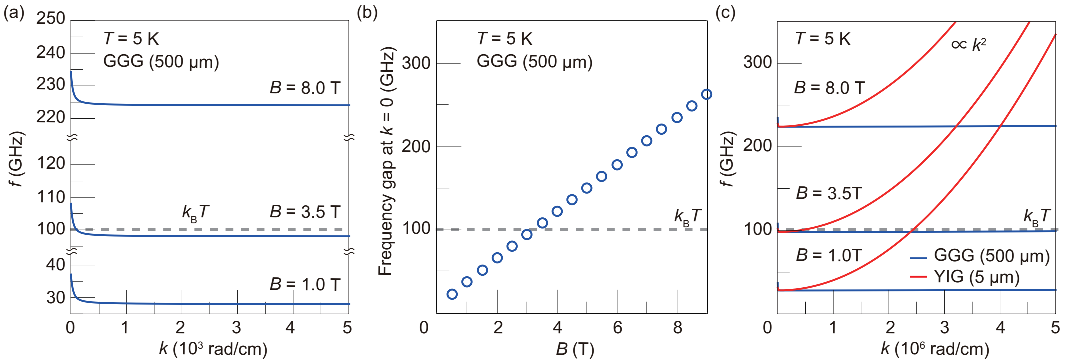


Supplementary Figure 11: Computed spin wave dispersions and gaps of GGG and YIG. **a,** Spin-wave dispersions of GGG (thickness = 500 μm) at $T = 5$ K at $B = 1$, 3.5, and 8 T for $k < 5 \times 10^3$ rad/cm. **b,** The spin-wave excitation gap of GGG at $k = 0$ at $T = 5$ K as a function of $B$. **c,** Comparison between the spin-wave dispersions of GGG (blue solid lines) and YIG (red solid lines, thickness = 5 μm) at $T = 5$ K at $B = 1$, 3.5, and 8 T for $k < 5 \times 10^6$ rad/cm. The dashed lines indicate $hf = k_B T$.

GGG (blue solid lines) at $T = 5$ K and several $B$, where the dashed lines indicate again $hf = k_BT$. At high temperatures, exchange spin waves with high group velocities are excited in YIG (the ~ $k^2$ term). Even at low temperatures, exchange waves play a role in YIG because they cause the up bending of the flat dipolar bands and generate a band minimum.

The frequency of the dominating spin waves in nonlocal transport is often referred to as $hf = k_BT$, but this is not precise. Spin waves (magnons) obey Bose-Einstein statistics for a certain temperature $T$ and chemical potential $\mu$, and the scattering times are frequency dependent. The expression of spin waves with $hf = k_BT$ rather emphasizes that the relevant spin waves (magnons) are thermally populated and at elevated temperatures far from the spin wave band minimum.

**Suppelemtary References**


1. Kajiwara, Y. *et al.* Transmission of electrical signals by spin-wave interconversion in a magnetic insulator. *Nature* **464,** 262–266 (2010).

2. Takahashi, S., Saitoh, E., & Maekawa, S. Spin current through a normal-metal/insulating-ferromagnet Junction. *J. Phys.*: *Conf. ser.* **200,** 062030 (2010).

3. Jia, X., Liu, K., Xia, K., & Bauer, G. E. W. Spin transfer torque on magnetic insulators. *Europhys. Lett.* **96,** 17005 (2011).

4. Barak, J., Huang, M. X. & Bhagat, S. M. Electron Paramagnetic resonance study of gadolinium -gallium-garnet. *J. Appl. Phys.* **71,** 849 (1992).

5. Takahashi, S. & Maekawa, S. Spin injection and detection in magnetic nanostructures. *Phys. Rev. B* **67**, 052409 (2003).

6. Cornelissen, L. J., Peters, K. J. H., Bauer, G. E. W., Duine, R. A., & van Wees, B. J. Magnon spin transport driven by the magnon chemical potential in a magnetic insulator. *Phys. Rev. B* **94,** 014412 (2016).

7. Bloembergen, N. On the interaction of nuclear spins in a crystalline lattice. *Physica* **15,** 386–426

(1949).

8. Cornelissen, L. J. & van Wees, B. J. Magnetic field dependence of the magnon spin diffusion length in the magnetic insulator yttrium iron garnet. *Phys*. *Rev*. *B* **93,** 020403(R) (2016).

9. Schmidt, G., Ferrand, D., Molenkamp, L. W., Filip, A. T., & van Wees, B. J. Fundamental obstacle for electrical spin injection from a ferromagnetic metal into a diffusive semiconductor. *Phys*. *Rev*. *B* **62,** R4790(R) (2000).

10. Cornelissen, L. J., Shan, L., & van Wees, B. J. Temperature dependence of the magnon spin diffusion length and magnon spin conductivity in the magnetic insulator yttrium iron garnet. *Phys*. *Rev*. *B* **94,** 180402 (2016).

11. Serga, A. A., Chumak, A. V., & Hillebrands, B. YIG Magnonics. *J. Phys. D: Appl. Phys.* **43,** 264002 (2010).
